# Phagocyte Responses to Protozoan Infection and How *Toxoplasma gondii* Meets the Challenge

**DOI:** 10.1371/journal.ppat.1002794

**Published:** 2012-08-02

**Authors:** Eric Y. Denkers, Anne G. Schneider, Sara B. Cohen, Barbara A. Butcher

**Affiliations:** Department of Microbiology and Immunology, College of Veterinary Medicine, Cornell University, Ithaca, New York, United States of America; University of Wisconsin Medical School, United States of America

## Introduction

The intracellular protozoan *Toxoplasma gondii* is arguably the most successful parasite on the planet. It exploits an uncommonly wide host range that encompasses essentially all warm-blooded animals including both mammalian and avian species. Sexual reproduction in the intestine of the definitive host, the cat, results in fecal shedding of up to 10^8^ highly infectious oocysts. The presence of felines from equatorial latitudes to sub-arctic regions of the globe ensures widespread distribution of the parasite. Moreover, unlike closely related apicomplexans such as the *Plasmodia*, passage through the definitive host is not obligatory to complete the life-cycle, because *T. gondii* can be transmitted from one intermediate host to the next through predation and carnivorism [1].

**Figure 1:**
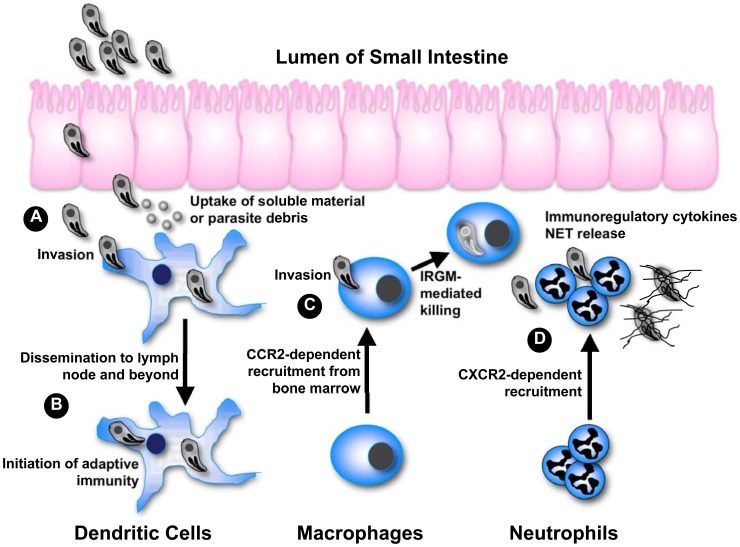
Integrating phagocyte function with *T. gondii* infection. (A) After crossing the epithelium of the small intestine, tachyzoites invade DC and stimulate IL-12 production through the activity of GRA5. Uptake of extracellular parasite debris containing profilin also stimulates IL-12 production. Some parasite strains trigger activation of STAT3 and STAT6 through secretory kinase ROP16, simultaneously down-regulating IL-12 induction. (B) Infected DC migrate to the draining lymph node and beyond, thereby disseminating infection. Infected as well as antigen-bearing DC also initiate immunity in the lymph node. (C) Inflammatory macrophages are recruited from the bone marrow in dependence upon CCR2. Following IFN-γ-mediated induction of IRGM-family effector molecules, intracellular parasites are destroyed by disruption of the parasitophorous vacuole. Some parasite strains release an effector kinase called ROP18 that phosphorylates and inactivates host IRGM molecules. (D) CXCR2-dependent neutrophils are also part of the innate response to *Toxoplasma*, although their functional importance is debated. In vitro studies have shown that neutrophils release several proinflammatory cytokines important for DC activation and resistance to the parasite. Neutrophils also produce extracellular traps that can ensnare parasites possibly interfering with invasion.

While *Toxoplasma* causes asymptomatic infection in most hosts, the parasite may emerge as an opportunistic infection under immunodeficient conditions such as in AIDS patients and during congenital infection. This danger underscores the importance of the encounter between *T. gondii* and the host immune system in determining the success of this particular host-parasite interaction. It is well understood that complete evasion of immunity (or for that matter passive failure to trigger immunity) results in rampant infection and host death, an outcome undesirable for both host and parasite. At the same time, we are learning in greater detail the mechanisms employed by the host immune system to destroy *Toxoplasma*. The parasite must obviously avoid this outcome of immunity to ensure persistence. The global success of *T. gondii* (over 10^9^ asymptomatic infections in humans alone) suggests that the parasite employs sophisticated molecular strategies to balance evasion versus activation of the host immune response. As summarized in Figure 1, the multiple ways this unifying principle plays out is revealed in studies on infection in phagocytes of innate immunity, namely dendritic cells (DC), macrophages, and neutrophils. These cells are among the first to encounter and be infected by *Toxoplasma* after the parasite crosses the intestinal epithelium. The studies together form a platform from which we can further understand the complex relationship between microbial pathogens and cells of the innate immune system.

## Dendritic Cells Are Sentinels and Trojan Horses

Early on it was recognized that DC were an early source of IL-12 driving protective Th1 responses to *Toxoplasma*. Further studies using an intraperitoneal infection model showed that ablation of CD11c^+^ DC results in failure to mount protective immunity and death during infection [2]. With the discovery of Toll-like receptors (TLR) and their ligands in the late 1990s, attention turned to the role of this system in sensing protozoan pathogens, in particular *T. gondii* [3]. Indeed, mice lacking MyD88, a central adaptor of TLR signaling, are extremely susceptible to infection. More specifically, it has been shown using cell-specific gene-deleted mouse strains that MyD88 expression in CD11c^+^ dendritic cells is required to resist *Toxoplasma* infection [4]. There is evidence for involvement of mouse TLR2, TLR4, TLR9, and TLR11 in the innate immune response to *T. gondii* [3]. Of these receptors, deletion of TLR11 has the most dramatic effect on loss of host resistance [5]. However, *Tlr11* ^−*/*−^ mice fail to recapitulate the extreme susceptibility phenotype of *Myd88* ^−*/*−^ mice. This has led to the suggestion that multiple TLR function together to provide optimal resistance, or alternatively that other untested TLR serve as the major MyD88-dependent receptor mediating protective immunity.

*Toxoplasma* profilin (TgPRF), an actin polymerizing molecule, is the ligand recognized by TLR11 [5]. In DC, TgPRF stimulates TLR11-dependent IL-12 production. Interestingly, it was recently found that this response occurs through phagocytic uptake of parasite material followed by TLR11 activation from within the endolysosome [6]. Surface-expressed glycosylphosphatidylinositol moieties purified from tachyzoites have also been found to mediate TLR2 and TLR4 activation [3], although the in vivo importance of this phenomenon is not clear. While the TLR11/TgPRF interaction is significant in the rodent response to *Toxoplasma*, the importance of TgPRF in human infection is uncertain since we do not express functional TLR11. In addition to TLR-dependent recognition of *Toxoplasma*, there is clear evidence for MyD88-independent resistance. This is because *Myd88* ^−*/*−^ mice develop strong (albeit delayed) Th1 responses during oral infection, and the same mouse strain develops protective immunity following intraperitoneal infection with attenuated parasites [7]. In this regard, it was recently shown that release of tachyzoite dense granule protein GRA5 into the host cytoplasm by intracellular parasites bypasses MyD88 to activate NFκB and stimulate IL-12 synthesis [8]. The relative roles that profilin and GRA5 assume during normal infection have not yet been determined. However, GRA5 IL-12 inducing properties are parasite strain-specific, in that only one lineage (Type II) of the three predominant strains found in Europe and North America possess this activity [8]. On the other hand, there is no evidence that profilin acts in a parasite-strain-dependent manner.

Another function of DC during the response to *Toxoplasma* is to serve as early reservoirs of infection [9], [10]. It has been suggested that parasites utilize DC in a “Trojan horse” strategy to disseminate throughout the host. Upon in vitro infection, DC acquire a hypermotility phenotype that is dependent upon host cell G-protein signaling triggered by the parasite. Intraperitoneal inoculation of tachyzoite-harboring DC spreads infection more rapidly than injection of extracellular parasites alone, suggesting that DC hypermotility promotes dissemination during in vivo infection, although whether a similar phenomenon occurs during oral infection is not clear [11]. Interestingly, hypermotility and in vivo dissemination of infected DC occur most efficiently with Type II *Toxoplasma*, the strain most frequently found in human infection [12].

## Macrophages/Monocytes Are Potent Killers but They Are Vulnerable to Exploitation

Activated cells of the macrophage/monocyte lineage have long been understood to possess potent microbicidal activity against *Toxoplasma* and other intracellular pathogens. CCR2-dependent inflammatory monocytes activated by IFN-γ and expressing Ly6C have recently been identified as important effectors of resistance to *Toxoplasma*. After oral infection, these cells are recruited to the intestinal mucosa, where they appear to control infection through direct parasite killing (Figure 1) [13]. In addition to their microbicidal activity, it is possible that they are involved in triggering Th1 immunity since inflammatory monocytes, like DC, also express IL-12 and TNF-α [13].

Killing of *Toxoplasma* in mice depends to a great extent on an immunity-related GTPase (IRG) protein-based resistance system [14]. The IRG proteins are strongly induced by IFN-γ, and they are sequentially loaded onto the cytoplasmic face of the parasitophorous vacuole membrane (PVM) that harbors intracellular tachyzoites. Through mechanisms not well understood, this results in disintegration of the PVM and release of tachyzoites into the host cell cytoplasm where they cannot survive. However, the IRG resistance system is inconsistently expressed across species and is in fact absent in humans [14]. Other important mechanisms of killing presumably exist. In this regard, it is of interest that human monocyte-derived macrophages kill intracellular *Toxoplasma* in an IFN-γ-independent, CD40-dependent mechanism that appears to involve autophagy [15].

The well-known fact that *Toxoplasma* strains differ in mouse virulence and the ability to perform genetic crosses in cats have allowed investigators to use quantitative trait locus analysis to identify virulence-associated parasite loci [16], [17]. Possibly indicating the importance of rodents in the natural life-cycle of *T. gondii*, the *Rop18* gene, encoding the secretory rhoptry protein kinase ROP18, was found to play a key role in inactivating IRG molecules, allowing *Toxoplasma* strains expressing active ROP18 to avoid killing by this resistance system [18], [19]. Along the same lines, ROP16 was identified as a parasite kinase injected into the host cytoplasm that activates signal transducer and activator of transcription (STAT) molecules through tyrosine phosphorylation [20]. Because both STAT3 and STAT6 are activated by ROP16, the effects of ROP16 on the host cell are complex and likely to involve both avoidance of killing and access to host cell nutrients [21], [22]. Furthermore, suppression of macrophage cytokines IL-12 and TNF-α are among the consequences of parasite-mediated STAT3 activation [23]. Recently, the parasite conoid-associated protein CAP-1 was identified using a forward genetics approach [24]. Deletion of this molecule increases the sensitivity of parasites to nitric-oxide-dependent killing, although how CAP-1 functions is not known. The widespread suppressive effects of *Toxoplasma* on macrophage responses has led to investigations on chromatin remodeling around promoters of IFN-γ and LPS responsive genes. There is evidence that *T. gondii* interferes with histone phosphorylation and acetylation at loci responsive to STAT-1 and TLR-mediated signaling [25], [26]. Because this phenomenon is strain independent, it seems unlikely to involve the polymorphic parasite kinases discovered through virulence locus identification. Regardless, targeting histone modification may be a way for the parasite to specifically affect the activity of large cohorts of host genes using a single mechanism.

## Neutrophils: Knowns and Known Unknowns

The standard view of neutrophils is that they rapidly home to sites of infection, phagocytose pathogens, release anti-microbial granules, and undergo apoptosis. However, neutrophils can also release immunoregulatory cytokines and chemokines, suggesting that they may also participate in shaping immunity [27]. There is evidence that neutrophils are important in recruiting and activating dendritic cells in response to microbial pathogens including *Toxoplasma* [28]. Recently, these cells have been found to release chromatin and granule-associated neutrophil extracellular traps (NET) that ensnare and kill microbes. Originally described as a response to bacterial and fungal pathogens, new studies indicate that NET release also occurs in response to protozoan pathogens, including *Toxoplasma* [29]. Both human and mouse neutrophils undergo a vigorous parasite strain-independent NET response during tachyzoite co-culture, and entrapment within NET could in principle interfere with the ability of *T. gondii* to find safe harbor within host cells.

Although neutrophils are recruited in abundance in response to *Toxoplasma* and their depletion is associated with increased susceptibility to infection, their in vivo function is controversial. Accumulation of these cells in the intestinal mucosa following infection could be a response to bacteria that translocate from the lumen to the subepithelium during *Toxoplasma* infection rather than a host response to the parasite itself [30]. It has been argued that neutrophils have no role in protection, at least in mouse models of infection [31]. The reason for the uncertainty is that in vivo antibody-mediated depletion protocols long used to remove neutrophils are now understood to also eliminate inflammatory monocytes because of common expression of Gr-1 (Ly6C/G). In fact, a recent depletion study using a neutrophil-specific anti-Ly6G antibody failed to find a protective effect of these cells on resistance to *Toxoplasma* infection [31]. Nevertheless, mice lacking CXCR2 fail to recruit neutrophils during *T. gondii* infection and display an increase in susceptibility, albeit to a lesser extent than removal of Ly6C/G^+^ cells [32]. This particular study was carried out with CXCR2 knockout mice on a BALB/c genetic background, unlike antibody depletion studies that were performed on a C57BL/6 background. It is possible that the role of neutrophils during *Toxoplasma* infection varies depending on mouse strain, host species, or parasite lineage.

## Conclusions

*Toxoplasma* and other tissue-invasive microbial pathogens encounter the innate immune system from the earliest stages of infection. Using genetically altered mice and parasites in combination, we are gaining fascinating insight into the molecular biology of the host-parasite interaction that plays out between *Toxoplasma* and the phagocytes of innate immunity. The overall theme emerging is that faced with a strong and multi-pronged host innate immune response, *Toxoplasma* does not retreat but actively engages cells of the innate defense system (Figure 1). Balancing stimulation of host defense with avoidance of immune elimination allows this extraordinary parasite to persist in its host and ensures widespread transmission throughout the vertebrate animal kingdom.
